# Assessing the Reusability of 3D-Printed Photopolymer Microfluidic Chips for Urine Processing

**DOI:** 10.3390/mi9100520

**Published:** 2018-10-15

**Authors:** Eric Lepowsky, Reza Amin, Savas Tasoglu

**Affiliations:** 1 Department of Mechanical Engineering, University of Connecticut, Storrs, CT 06269, USA; eric.lepowsky@uconn.edu (E.L.); reza.amin@uconn.edu (R.A.); 2 Department of Biomedical Engineering, University of Connecticut, Storrs, CT 06269, USA; 3 Institute of Materials Science, University of Connecticut, Storrs, CT 06269, USA; 4 Institute for Collaboration on Health, Intervention, and Policy, University of Connecticut, Storrs, CT 06269, USA; 5 The Connecticut Institute for the Brain and Cognitive Sciences, University of Connecticut, Storrs, CT 06269, USA

**Keywords:** microfluidics, 3D printing, reusability, biofouling

## Abstract

Three-dimensional (3D) printing is emerging as a method for microfluidic device fabrication boasting facile and low-cost fabrication, as compared to conventional fabrication approaches, such as photolithography, for poly(dimethylsiloxane) (PDMS) counterparts. Additionally, there is an increasing trend in the development and implementation of miniaturized and automatized devices for health monitoring. While nonspecific protein adsorption by PDMS has been studied as a limitation for reusability, the protein adsorption characteristics of 3D-printed materials have not been well-studied or characterized. With these rationales in mind, we study the reusability of 3D-printed microfluidics chips. Herein, a 3D-printed cleaning chip, consisting of inlets for the sample, cleaning solution, and air, and a universal outlet, is presented to assess the reusability of a 3D-printed microfluidic device. Bovine serum albumin (BSA) was used a representative urinary protein and phosphate-buffered solution (PBS) was chosen as the cleaning agent. Using the 3-(4-carboxybenzoyl)quinoline-2-carboxaldehyde (CBQCA) fluorescence detection method, the protein cross-contamination between samples and the protein uptake of the cleaning chip were assessed, demonstrating a feasible 3D-printed chip design and cleaning procedure to enable reusable microfluidic devices. The performance of the 3D-printed cleaning chip for real urine sample handling was then validated using a commercial dipstick assay.

## 1. Introduction

There is significant interest in microfluidics due to its large variety of applications, including cancer screening [1,2,3], micro-physiological system engineering [4,5], high-throughput drug testing [6,7], and point-of-care diagnostics [8,9,10,11,12,13]. In particular, point-of-care devices can help to enable routine health monitoring and preventative care, which can improve public health while increasing healthcare savings. In a study reported by Health Affairs, it has been shown that a 90% increase in specific preventative screenings back in 2006 would have saved more than 2 million lives without a significant increase in healthcare costs—in fact, a 0.2% decrease in costs was estimated [14]. Microfluidic devices have a proven record of being effective analytical devices, capable of controlling the flow of fluid samples, containing reaction and detection zones, and displaying results, all within a compact footprint, and are therefore appropriate for addressing this need for routine and preventative care [15,16,17,18,19,20].

While microfluidic devices are low-cost in terms of material cost, conventional fabrication is challenging and costly. Furthermore, there is an unmet need to address the convenience of implementing microfluidic devices in sophisticated medical devices: users should not need to replace the chips routinely. To address the burdensome and expensive fabrication of microfluidic devices, as well as the need for convenience with respect to medical devices, there is an increasing need to develop microfluidic devices for long-term use [21,22,23,24]. While single-use microfluidic chips may be practical for some applications, such as for prototyping and experimental testing, devices that allow for multiple uses are better suited for high-throughput testing and point-of-care diagnostics, as the limited lifetime of microfluidic devices has been cited as a barrier to commercialization [25]. Furthermore, market trends indicate that by 2018, the field of microfluidics was estimated to reach $3.6–5.7 billion [26], demonstrating the continued growth of the field, particularly within clinical diagnostics, pharmaceutical research, point-of-care diagnostics, and analytical applications. Thus, multiuse devices will prove very useful while combatting the waste that accumulates with disposable chips in these applications. For these reasons, the reusability of conventional poly(dimethylsiloxane (PDMS) microfluidic devices has previously been assessed [24].

Microfluidic devices are traditionally fabricated by combining photolithography techniques for making a master mold and soft lithography using PDMS and bonding. The process as a whole is complex, expensive, laborious, as well as time-consuming [27]. There exist several alternative approaches, one of which being 3D printing. Using hydrogels, Beebe et al. developed an ultrafast fabrication platform combining liquid-phase polymerization and lithography compatible with a variety of geometries and valve designs [28]. Another alternative is Teflon, which was demonstrated by Rolland et al. [29]. Unlike PDMS, the liquid-Teflon, a type of photocurable perfluoropolyether (PFPE), is resistant to swelling in common organic solvents, in addition to being liquid at room temperature and exhibiting low surface energy, low modulus, high gas permeability, and low toxicity. Glass can also be used for microfluidic device fabrication. For instance, femtosecond laser direct writing can modify the interior of glass through multiphoton absorption to form microfluidic channels and optofluidic components [30]. Femtosecond laser processing can even be used to fabricated ultrathin, flexible microfluidic chips [31]. Microfluidic devices can also be fabricated using plastics. A study by Wan et al. indicated that poly(methyl methacrylate) (PMMA) can be recycled for biological microfluidic device applications. In this experiment, researchers pumped bleach and ethanol through the microfluidic channels after the chip was used, and then they melted the PMMA so that the plastic could be reused for multiple chip iterations [32]. While studies have indicated that the recycling of plastic-based microfluidic chips is possible, there is a lack of research being conducted on the reusability of microfluidic chips. Reusability is advantageous over recyclability since recycling involves the remanufacturing of the device. Investigation is needed to determine if merely rinsing the channels with a cleaning agent, such as a phosphate-buffered solution (PBS) or deionized (DI) water, will allow for the microfluidic chip to be reused with negligible protein absorption and cross-contamination between samples.

3D printing is a new technique that can bypass many limitations seen with PDMS-based approaches, such as expensive equipment and clean room facilities, thus making the fabrication process easier, cheaper, and more flexible [26,27,33,34]. There are various 3D printing techniques that can be used to fabricate microfluidics. The most applicable ones to microfluidics are stereolithography (SLA), multijet modeling (MJM), and fused deposition modeling (FDM) [27,33,34,35]. All techniques use a computer-aided design (CAD) sketch as a blueprint and build the structure layer-by-layer from printing material, which can be plastic filaments, liquid resin, or powder [27,36]. The most commonly used commercialized 3D printing technology for micro-fabrication is the SLA technique, which is defined as a method for making solid objects by successively printing thin layers of a curable material (i.e., a UV-curable liquid resin) on top of the other [35]. In SLA printing, a laser beam or projector is used to crosslink the resin in the predetermined pattern [33]. For microfluidic devices, a microchannel is built by photo-polymerizing the channel walls and then draining the uncured resin from the channel cavity after the printing is complete [37]. For the purpose of microfluidic devices which involve imaging, clear resin is available.

When a microfluidic device is first manufactured and put into use, it is assumed that the channels will provide a clean environment for the samples to flow through. However, the size and composition of these devices can make it unusable after just a few experiments if not designed with reusability in mind [38]. Within the channels of a microfluidic device, the surface area-to-volume ratio is high, meaning more of the fluid is exposed to the surfaces of the channel per unit volume. If the device is made of a porous or hydrophobic material, it can allow molecules to stick to the channel walls, rendering it unusable. In addition, the charge on a molecule can cause them to stick to the channel walls if the chip is made of a polar material [39]. Further, if there are areas in the channel where the fluid flow becomes static, especially around channel intersections, there is a high likelihood that a molecule buildup will occur, causing elevated levels of absorption or contamination by the molecule, or even reducing or completely inhibiting the flow through that channel [38]. Nonetheless, the change in flow through the channel should not overshadow the larger issue: the buildup of molecules, and possibly cells, can cause largely inaccurate results in sample testing. A primary challenge in producing microfluidic devices for long-term use is the biofouling that often occurs on the surface of integrated channels and features [39,40]. This phenomenon occurs, due to surface interactions between the walls of the channels and the biological sample flowing through the channels. To address the long-term use of microfluidic devices, there has been a growing research interest in developing new anti-fouling methods and materials [21,22,41,42,43]. Additionally, other alternatives, such as recyclable chips or reusable chips, are actively being explored [23,24,32,44].

**Figure 1:**
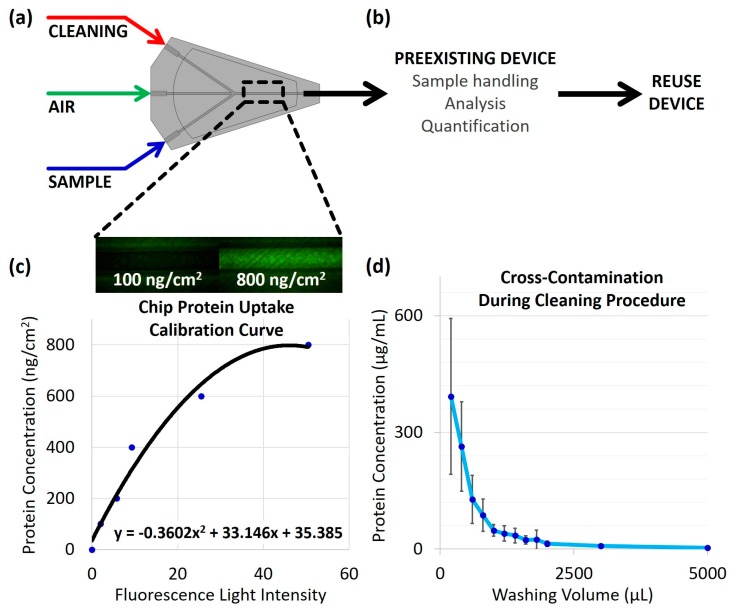
Design, core functionality, and experimental overview: (**a**) Generalized design of cleaning chip. The cleaning chip device consists of inlets for the sample, cleaning solution, and air, all connected to a single outlet. This design allows for a cleaning procedure to be performed on the chip itself and any external microfluidic device that is connected to the cleaning chip’s outlet; (**b**) Core functionality of the cleaning chip. The cleaning chip may be used in conjunction with a pre-existing microfluidic device, which may itself perform sample handling, analysis, quantification, etc., in order to render the external device a reusability microfluidic device by connecting the outlet of the cleaning chip to the inlet of the pre-existing device; (**c**) Experimental preview of chip protein uptake. The 3D-printed cleaning chip was connected to a syringe pump, and the cleaning solution was collected from the outlet of the cleaning chip for protein cross-contamination quantification, while the chip itself was assessed for protein absorption. A fluorescence detection method was used to determine the concentration of protein present in the collected samples from the outlet and within the chip itself; the CBQCA assay used fluoresces green by excitation with blue light. Above, the two images shown provide an example of the fluorescent images with their respective protein concentrations. Below, the calibration curve used for determining protein uptake by the chip was constructed by injecting known concentrations of protein into channels of a chip and imaging the channels using a fluorescence microscope, where the raw light intensity (on a scale from 0 to 255) was used as the independent variable; (**d**) Experimental preview of cross-contamination results. Collected samples were quantified by using a plate reader to measure the fluorescence of the cleaning solution from the outlet. The observed trend showed the decreasing protein concentration with the increasing washing volume.

Leveraging the advantages of 3D printing with the aim of addressing the need for reusable microfluidic devices due to their potential societal impact, herein, a 3D-printed microfluidic chip was designed to assess the reusability of 3D-printed chips by means of quantifying its absorption characteristics. The proposed solution serves to validate the ability to deliver a sample and clean the device for reusability. The core function of the demonstrated 3D-printed microfluidic cleaning chip is to integrate a cleaning procedure into a pre-existing single-use device such that the chip/device may be reused (Figure 1b). To facilitate the cleaning process, the cleaning chip is connected to a syringe pump to deliver either the sample, cleaning solution, or air, at a controlled rate, depending upon which inlet the pump is attached to; the inlet channels converge to a single outlet. In a real-world application, the sample would flow from the outlet of the cleaning chip into the sample handling portion of the pre-existing device (Figure 1a,b). For the purpose of assessing the protein uptake by the chip, a calibration curve (Figure 1c) was created by injecting protein samples of known concentration into channels of equal dimensions to the cleaning chip, performing fluorescent assays, and using a fluorescence microscope to quantify the fluorescent light intensity, in which a greater light intensity corresponds to a greater concentration of protein present. Specifically, the 3-(4-carboxybenzoyl)quinoline-2-carboxaldehyde (CBQCA) fluorescence detection method was used, in which the CBQCA fluoresces green in the presence of protein when excited by a blue light source. For assessing the cross-contamination between samples, the sample from the outlet was collected, CBQCA was added to the samples, and a plate reader was used to quantify the fluorescence, thereby reflecting the amount of protein present in the samples. By performing the CBQCA assay on samples over time during the cleaning procedure, the cross-contamination as a function of washing volume was determined (Figure 1d).

## 2. Materials and Methods

### 2.1. Design and Fabrication of 3D-Printed Microfluidic Cleaning Chip

**Figure 2:**
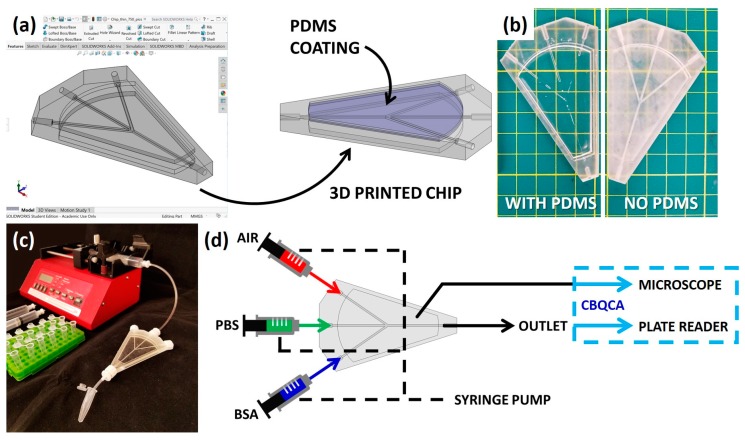
Overview of fabrication and experimental methods: (**a**) Microfluidic chip is first designed in SolidWorks, and then 3D-printed using a Formlabs Form 2 printer. Post-printing, a thin layer of PDMS is poured and cured on both faces of the chip; (**b**) Addition of the PDMS coating on the outside of the chip improves visibility. See a comparison of the chip with PDMS (on the left) to the chip without PDMS (on the right); (**c**) Image of experimental setup, showing the 3D-printed chip connected to a syringe pump. The sample from the outlet of the chip was collected in microcentrifuge tubes for fluorescence detection analysis; (**d**) Schematic representation of the experimental design and implementation of the 3D-printed microfluidic chip. The concentrated BSA solution, and phosphate buffered solution (PBS) as a cleaning solution, and air each have designated inlets which all converge to a single outlet. The sample from the outlet, collected at set intervals, and the chip itself are then analyzed for the protein concentration by the CBQCA quantitation method using a plate reader and microscope, respectively.

Microfluidic cleaning chips were designed using SolidWorks CAD modeling software (SolidWorks 2017–2018, Dassault Systèmes SOLIDWORKS Corp, Waltham, MA, USA), as seen in Figure 2a. The chip consisted of four channels: a biological sample inlet, a cleaning solution inlet, an air inlet, and a universal outlet. For each iteration of the chip, the outer dimensions were slimmed down to reduce material waste, and a viewing window was added to the top and bottom of the chip to improve through-chip visibility, while the channel width was held constant at 1000 µm. The CAD models were then fabricated using the Formlabs Form 2 3D SLA printer (Form 2, Formlabs Inc., Somerville, MA, USA). This 3D printer was chosen due to its high-resolution capabilities. Furthermore, clear resin was used to fabricate the chips, so it would be possible to view the channels externally (Clear Resin GPCL04, Formlabs Inc., Somerville, MA, USA). The clear resin also allowed for fluorescence readings to be taken through the chip. After 3D-printed chips were fabricated, 1 mL of PDMS (Cell Guard Encapsulation Kit, ML Solar, Campbell, CA, USA) was deposited to the viewing windows on each side of the chip in order to improve clarity by filling pores of the surface roughness of the 3D-printed material (Figure 2a,b). The PDMS was degassed and then baked for 20–25 min at 80 °C. The refractive indices of PDMS and the clear resin have been reported as 1.4 and 1.5, respectively [45,46]. For reference, the refractive index of mineral oil, which is often used to improve optical clarity of a rough surface, is around 1.47; however, the bottom viewing window of the 3D-printed chip would necessitate the mineral oil-coated surface to be overturned, which could pose a risk to the microscope used for protein quantification [47]. Due to the very similar refractive indices of the PDMS and 3D-printed material used in the fabrication of the chip, there was negligible light interference at the interface between the two materials.

To determine the optimum channel height for the microfluidic chip, dimensions ranging from 100 µm to 1000 µm were tested. Printing success was assessed by measuring the length of each open channel and comparing this to the expected channel length. Using 500 µm as a benchmark channel height, which printed semi-reliably and of which success depended greatly on printing parameters, the ideal printing orientation was determined by analyzing the printing success, as described above, in relation to print orientation with respect to the build platform of the 3D printer. Ultimately, the chips were printed with an angled vertical orientation, such that the channels could self-drain excess resin during printing. Additionally, the Form 2 had higher resolutions in the x- and y-directions than in the z-direction. The highest-resolution layer height, 0.025 mm, was used; in the x- and y-directions, the laser could fabricate lines down to 140 microns and holes down to 25 microns. By orienting the chips vertically, the smallest feature size (the channels) benefited from the higher resolution of the printer. Furthermore, by angling the chips, the cross-sectional area of the features was effectively increased. The final chip design was assessed for printing accuracy by measuring the width of the printed channels using a desktop microscope and comparing this to the expected 1000 µm width.

### 2.2. Cleaning Procedure

The 3D-printed cleaning chip was designed to enable the reusability of microfluidic chips by implementing a simple cleaning procedure to reduce biofouling and cross-contamination between the samples. The cleaning procedure utilized a syringe pump and the four channels of the cleaning chip (Figure 2c,d). First, the biological sample inlet was used: a representative protein sample, 2 mL of bovine serum albumin (BSA) at a high concentration (20 mg/mL), was pumped through the chip, with the cleaning solution and air inlets sealed, using a syringe pump set to 1 mL/min (Thermo Fisher Scientific, Waltham, MA, USA). Following the BSA, air was pumped from the air inlet and through all three non-air channels individually by sealing the remaining two channels, with 0.5 mL of air for each channel, also set at 1 mL/min. The cleaning agent was then pumped, with the biological sample and air inlets sealed, for a total of 10 mL at 1 mL/min. Pure PBS and DI water were tested as the cleaning agent (Thermo Fisher Scientific, Waltham, MA, USA); PBS was determined to be the superior choice. The cleaning procedure ended with a second round of the air to dry all the channels of the chip.

### 2.3. Protein Cross-Contamination Quantification

While a cleaning agent was pumped through the chip during the cleaning procedure, 200 μL samples from the outlet were collected at 13 intervals: 200, 400, 600, 800, 1000, 1200, 1400, 1600, 1800, 2000, 3000, 5000, and 10,000 μL. The protein level of these samples passing through the device was quantified using a CBQCA fluorescence detection method (CBQCA Protein Quantitation Kit (C-6667), Molecular Probes, Inc., Eugene, OR, USA). Using this method, BSA protein as little as 10 ng can be detected. In the presence of protein, the CBQCA fluorescence green when excited by blue light. To measure the cross-contamination level of the cleaning solution after passing through the system, 5 μL of KCN (20 mM) and 10 μL of CBQCA reagent (5 mM) were added to 135 μL of each sample in a microplate. After allowing the samples in the microplate to incubate for 1 hour in darkness, the fluorescence level was measured using a BioTek Synergy H1 plate reader (Synergy H1 Hybrid Multi-Mode Reader, BioTek, Winooski, VT, USA).

### 2.4. Protein Uptake by Cleaning Chip Calibration and Quantification

In order to quantify the protein uptake by the cleaning chip, a calibration chip consisting of 10 channels of equal width and height as the channels of the cleaning chip was designed and 3D-printed. Each channel was filled with 135 μL of a BSA-in-PBS solution of known concentrations (1, 0.5, 0.25, 0.1, 0.08, 0.06, 0.04, 0.02, 0.01, and 0 mg/mL) mixed with 5 μL of KCN (20 mM) and 10 μL of CBQCA reagent (5 mM). After incubating for 1 hour in darkness, the channels of the chip were imaged using a Zeiss Observer z1 fluorescence microscope (Carl Zeiss Microscopy GmbH, Jena, Germany) on the 2.5x objective. The greater the concentration of protein present, the greater the intensity of the green fluorescence. Intensity was measured using ImageJ (v1.48k, National Institutes of Health, Bethesda, MD, USA).

The protein absorption by the chip—the same chip as the cross-contamination experiment after being exposed to the 20 mg/mL BSA solution—was then characterized by adding a mixture of 135 μL of PBS, 5 μL of KCN (20 mM), and 10 μL of CBQCA reagent (5 mM) to the chip’s channels. After a 1-hour incubation period in darkness, the channels of the chip were imaged using the fluorescence microscope. The light intensity of the channels was compared to the intensity of the calibration chip to determine the amount of protein absorbed by the chip. The protein uptake was quantified for four cases: a single-use chip with DI, a single-use chip with PBS, a 6-cycle chip with PBS, and a 6-cycle chip with PBS and a post-cleaning treatment of 10 mL of 0.25% trypsin (Trypsin-EDTA (0.25%), phenol red, 25200-056, Thermo Fisher Scientific, Waltham, MA, USA). Trypsin is a protease which hydrolyses other proteins, and therefore reduces the levels of protein uptake by the cleaning chip significantly by digesting the adhered and absorbed proteins.

### 2.5. Longitudinal Protein Cross-Contamination

The longitudinal sample protein cross-contamination was characterized by repeatedly performing the same analysis as for protein cross-contamination. After completion of each round of pumping samples through the chip, the chip was cleaned following the prescribed cleaning process. This procedure of flowing the biological/protein sample, collecting the PBS samples from the outlet, and cleaning the chip was repeated to demonstrate the collective contamination as a result of repeated use of the chip.

### 2.6. Protein Quantification for Urine Processing

The reusability of the 3D-printed cleaning chip for real urine sample handling was validated by assessing the protein cross-contamination between samples and the protein uptake by the chip. Urine samples were collected from volunteers according to protocol # H17-043, approved by the University of Connecticut Institutional Review Board. All subjects provided informed consent. For studying cross-contamination, samples were pumped through the microfluidic device and collected in microcentrifuge tubes. Commercially available urine analysis dipsticks (Urinalysis Reagent Strips, HealthyWiser LLC., Los Angeles, CA, USA) were then dipped into the collected samples and allowed to incubate for 60 seconds before being imaged with a DSLR camera (REBEL T6, Canon, Melville, NY, USA) in the RAW image mode with flash for consistent lighting. The captured images were then converted to grayscale, such that the light intensity of the grayscale image could be correlated to a specific protein concentration. To facilitate this quantification, a calibration curve was constructed by least-squares curve fitting of the light intensities from images of the dipstick protein assay pads for various BSA concentrations (0, 0.15, 0.3, 1, 3, 10, and 20 g/L).

The cross-contamination was studied for an “extreme-case” scenario, in which pumped samples alternated between urine and high-concentration (20 g/L) BSA. A modified cleaning procedure was performed between each sample: in addition to pumping PBS (the cleaning solution) through the universal exit channel, PBS was also pumped through the sample inlet channel. For each cycle, four samples were collected and analyzed using the dipstick assay: urine, post-urine PBS, BSA, and post-BSA PBS. The protein uptake by the chip was also studied for this “extreme-case,” in addition to exposure to pumped urine samples for 30 and 90 min. The total sample volume of the “extreme-case” was approximately equal to the volume of the 30-min exposure to urine. For each of the three urine experiments, protein uptake by the chip was quantified using the previously described CBQCA fluorescent detection method and fluorescent microscope.

## 3. Results

### 3.1. Design and Fabrication of 3D-Printed Microfluidic Cleaning Chip

An early chip design (the top-most design depicted in Figure S1a in Supplementary Materials) was fabricated with a 500 µm channel height by 3D printing at different orientations. That is, the chip was oriented, with respect to the print platform, at the (Formlabs Preform software, v2.17.1, Formlabs Inc., Somerville, MA, USA) default 45-degree angle, vertically, horizontally, and lied flat flush along the print platform. The channels of the printed chips were then injected with food dye to visualize the printed length of each channel. The printed length (i.e., the length colored by the food dye) was measured and reported as a fraction of the total expected length; these results are presented in Table 1. The outlet channel consistently had the lowest printing success, attributed to the fact that the outlet was the furthest from the print platform. As the chip was printed, the distance from the print platform increased, allowing for greater deflection that results in misalignment and misprints. The default orientation was chosen as the optimal orientation, despite the vertical orientation having slightly better success, since the default angled orientation allowed for a greater amount of supports. The final chip design (Figure S1b, Supplementary Materials) was also assessed for printing accuracy. A desktop microscope was used to image the width of the channels. The measured printed width and the percent error are reported in Table 2. It should also be noted that in both Table 1 and Table 2, the left inlet exhibited greater printing success than the right inlet; this can be attributed to the slight rotation about the vertical axis used by the default orientation setting, which positions the left inlet was marginally closer to the print platform than the right inlet. The low average percent error in printing was deemed sufficient to not warrant a redesign of the chip’s channels.

The progression of the chip design process is depicted in Figure S1a (Supplementary Materials), from top to bottom. The initial chip was created with a specific focus on developing the interior channel pattern, as well as the channel inlet/outlet ports along the perimeter of the chip. As the design iterations continued, changes were made to the outer dimensions of the chip, reducing material usage and print time by removing excess solid portions (Figure S1e, Supplementary Materials). In addition, a viewing window was added, first to the top only and then to both faces of the chip, to increase through-chip visibility, which is particularly important for performing fluorescence imaging on the chip. The final chip design is presented in Figure S1b (Supplementary Materials), which features large viewing windows and an overall slim design with channels of 1000 µm wide by 750 µm high. To determine the ideal channel height, a parallel channel chip was designed, as seen in Figure S1c (Supplementary Materials), where each channel had a constant width of 1000 µm and the height was varied for 100, 200, 300, 400, 500, and 1000 µm. By eliminating channels that did not print (i.e., the channel was filled with cured resin), it was deduced that the optimal channel height was between 500 µm and 1000 µm. The final chip design was then printed with 500 µm, 625 µm, and 750 µm channel heights, and 750 µm was ultimately the dimension with the best and most reliable printability. Using the final channel dimensions, a calibration chip was printed (Figure S1d, Supplementary Materials), which consisted of ten channels; each channel was then filled with different concentrations of BSA and imaged using the fluorescence microscope.

### 3.2. Characterization of Protein Cleaning Procedure

**Figure 3:**
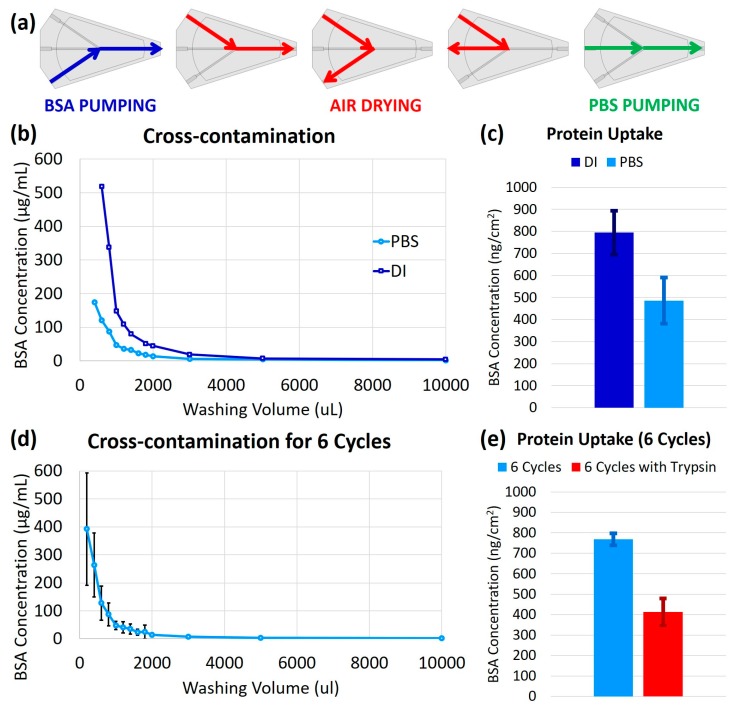
Protein cleaning characterization: (**a**) Graphical representation of one-cycle of the cleaning procedure. The PBS from the outlet was collected at set intervals for the cross-contamination results; (**b**) Cross-contamination of a single-use chip using deionized (DI) water compared to PBS as a cleaning solution with the BSA concentration in the output PBS or DI sample using a new chip. Cross-contamination is significantly reduced by using PBS, as compared to DI; (**c**) Protein absorption by a single-use 3D-printed chip after cleaning with DI compared to PBS. After cleaning with PBS, approximately half as much protein uptake remained, as compared to after cleaning with DI; (**d**) Cross-contamination of a repeated-use chip with the BSA concentration in the output PBS sample for 6 cycles using the same chip repeatedly, graphed as the mean with standard deviation error bars; (**e**) Protein absorption by the 3D-printed chip after 6 cycles of repeated use. By adding a post-cleaning treatment of trypsin after the 6 cycles of repeated use, the protein uptake is decreased by nearly half.

The effectiveness of the cleaning process (explained graphically in Figure 3a) for reducing the degree of cross-contamination between samples and the protein absorption by the chip was assessed using CBQCA assay for various scenarios: after one cycle use of the chip with DI water as the cleaning agent (i.e., the BSA sample was applied and the channels were dried and washed with the cleaning agent), after one cycle use of the chip with PBS as the cleaning agent, after six cycles of repeated use of a chip with PBS as the cleaning agent, and a repeat of the six cycles of repeated use with PBS and an additional post-treatment of trypsin. For each experiment, 13 samples were collected from an outgoing cleaning agent at different time/volume points, as described above in Methods. Two cleaning agents, PBS and DI water, were used separately for comparison. For both of the cleaning reagents, the BSA concentration is high at first and then decreases with the more washing volume; this decreasing BSA cross-contamination trend follows an exponential decay as a function of washing volume. A negligible amount of BSA was seen in PBS samples after 3000 µL, while this value was 5000 µL for DI water (Figure 3b). These results showed that using PBS as a cleaning agent is over 4 times more effective than using DI water, in terms of cross-contamination.

In addition to cross-contamination, the amount of protein absorption by the chip was measured after one-cycle use and repeated cycles of sample introduction and cleaning. The fluorescent microscope was used to measure the amount of protein in the channels after adding CBQCA. The light intensities in the images were analyzed for each channel. The light intensities inside and outside the channel were calculated using ImageJ software (v1.48k, National Institutes of Health, Bethesda, MD, USA). The light intensity outside the channel was used as a control as there was no protein, and hence no illumination occurred in that area. Figure 3c shows the average amount of protein absorption after cleaning with PBS and DI water. The chip that was cleaned with DI water shows significantly more absorbed BSA than the chip cleaned with PBS, (794 ± 99) ng/cm^2^ compared to (486 ± 104) ng/cm^2^, respectively. This leads to the conclusion that cleaning with PBS is over 1.6 times as effective as cleaning with DI water, in terms of chip protein uptake. Therefore, in further experiments, PBS was chosen as the cleaning reagent.

The longitudinal cross-contamination was also investigated to validate the proposed 3D-printed microfluidic chip and cleaning process for application in a setting, where a single device could be used repeatedly. After flowing the BSA sample through the inlet, the channels were dried and washed by flowing air and PBS through the chip. This cycle was repeated six times, with the 13 samples collected for each cycle, and all the collected samples were analyzed with the CBQCA assay. Comparing the obtained plate reader fluorescence results, it was clear that the amount of protein in the collected samples is reduced by increasing the amount of washing volume (Figure 3d). The average BSA concentration in the first 200 µL of PBS collections of all cycles was (392 ± 200) µg/mL (standard deviation is depicted in Figure 3d using error bars) and this amount is decreased sharply within 1000 µL of washing with PBS and then dwindled to zero. The overall trend is similar to the results of one-cycle cross contamination assessment. As seen in Figure 3d, the standard deviation in the measured BSA concentration for the first four samples of each cycle is significant due to the accumulation of BSA in the chip after each cycle; in other words, the large error bars represent the variation between the increased number of cycles. For example, higher cycles would lie closer to the top of the error bars, while the beginning cycles would be towards the bottom. The error bars shorten afterwards, which demonstrated that no matter how many times the chip was used, after washing with as much as 5000 µL, all the possible BSA has been removed, or only negligible BSA is present afterwards. It should be noted that it does not mean that all the BSA has been washed out, as some of the BSA was absorbed and accumulated in the chip.

The protein uptake after six repeated cycles was shown to be (758 ± 30) ng/cm^2^ (Figure 3e), which is approximately twice the amount of uptake after single use of the chip. This showed a promising trend that even after six repeated cycles of use, chip protein uptake less-than doubles, demonstrating a favorable relationship in that there was not a one-to-one relationship between cycles (and therefore exposure to BSA sample) and protein uptake. This can be taken to mean that the total protein uptake by the chip may approach a plateau after a relatively low number of cycles of use. Furthermore, by flowing 10 mL of trypsin after the six cycles of samples and cleaning, the protein uptake was reduced to (412 ± 66) ng/cm^2^. These favorable results are due to the protease function of the trypsin, which digests a portion of the BSA on the chip. This means that by adding trypsin, the effectiveness of the cleaning procedure can be further improved.

### 3.3. Protein Quantification for Urine Processing

**Figure 4:**
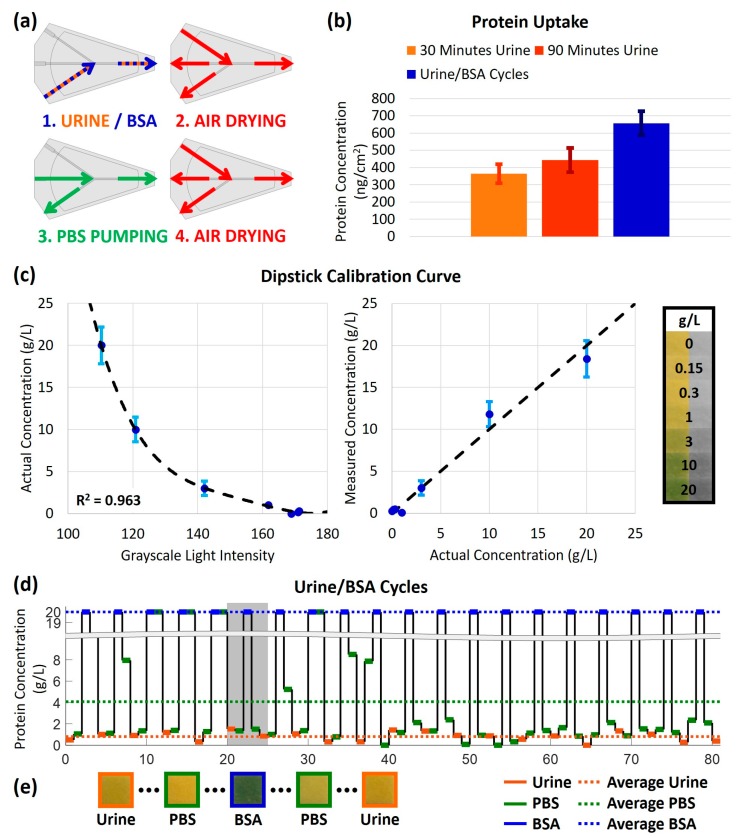
Performance of 3D-printed cleaning chip for urinary protein measurement: (**a**) Graphical representation of one-cycle of the modified cleaning procedure; (**b**) Protein absorption by the 3D-printed cleaning chip after 30 min of pumping urine (without cleaning), 90 min of pumping urine (without cleaning), and 20 cycles of alternating urine and high-concentration BSA samples (with cleaning between samples); (**c**) Left: calibration curve for the actual protein concentration versus the grayscale intensity of protein assay pads, calculated by least-squares curve fitting. Right: comparison of the calculated protein concentration using the calibration curve versus the actual concentration of the samples. Far right: photographs of the protein assay pads for 0, 0.15, 0.3, 1, 3, 10, and 20 g/L, shown in full color and grayscale; (**d**) Longitudinal protein concentration measurement for 20 cycles (81 pumping steps) of urine, PBS cleaning, high-concentration BSA, and a second round of PBS cleaning. Urine samples, PBS and BSA are represented by blue, green, and blue lines, respectively. Dotted lines represent the average protein concentration for the respective sample; (**e**) Photographs of the protein assay pads for Cycle 6, Steps 21–25.

The 3D-printed cleaning chip was demonstrated for application to urine testing using commercially available dipstick protein assay pads. For handling multiple samples, the cleaning procedure, depicted graphically in Figure 4a, was slightly modified to include PBS cleaning of both the sample inlet channel and the universal outlet channels. Additionally, the volume of cleaning solution was reduced to 300 µL. Using this modified cleaning procedure, urine samples and high-concentration (20 g/L) BSA were alternatingly pumped through the chip. The resulting protein uptake by the chip was (657 ± 69) ng/cm^2^ (Figure 4b). Comparatively, the protein uptake after 30 min of pumping urine (approximately the same total sample volume) with no intermediate cleaning was (364 ± 54) ng/cm^2^. The difference between these values is attributed to the high-concentration BSA, which was used in the former experiment. When the exposure time to urine without cleaning was increased to 90 min, the protein uptake was (443 ± 70) ng/cm^2^. This is a favorable result because for tripling the exposure time to urine, and tripling the volume of urine processed by the chip, the protein uptake only increased by 21.7%.

The cross-contamination between samples was also studied for the alternating urine and high-concentration (20 g/L) BSA. The protein assay pads of commercially available dipsticks were dipped in BSA samples of various concentrations (0, 0.15, 0.3, 1, 3, and 10 g/L). The resulting color change was converted into a change in grayscale light intensity. A calibration curve was created using least-squares curve fitting; the resulting curve, depicted on the left of Figure 4c, was found to be $y=-0.000002618x^{4}+0.001382x^{3}+0.2630x^{2}+20.8829x-552.0138$ with a correlation coefficient of $R^{2}=0.963$. The calibration curve was then verified by comparing the protein concentration calculated via image analysis to the actual protein concentration of the samples, as shown on the right of Figure 4c.

Figure 4d shows the longitudinal quantification of protein concentration for 20 cycles of urine samples, post-urine PBS cleaning solution, BSA samples, and post-BSA PBS cleaning solution (a total of 81 pumping steps). The average protein concentration in the urine samples after pumping through the cleaning chip, represented by the orange dotted line, was 0.8072 g/L, as compared to the true value of 0.4958 g/L. This deviation is attributed to the low sensitivity of the urine dipstick assay for low protein concentrations; in fact, for this range of protein concentration, the color key provided by the dipstick manufacturer only discerns between 0.3 g/L and 1.0 g/L. The average protein concentration of the PBS, represented by the green dotted line, was 4.0715 g/L. Although there is no protein present in the PBS cleaning solution originally, the protein concentration after pumping through the chip is elevated as it washes out protein from the previous sample. Despite the cross-contamination between the high-concentration BSA and subsequent PBS cleaning solution, the cleaning and air-drying steps are sufficient for yielding consistent urine concentration results. Figure 4e shows images of the dipstick pads for the gray region of Figure 4d.

## 4. Discussion

Reusability of microfluidic devices is critical for any devices that will go to market; however, fouling of the devices’ channels can pose a significant challenge. For a microfluidic device capable of being commercialized, it must work reliably over a long period of time and for a wide variety of samples [38]. Characterization of the protein absorption of 3D-printed material, specifically Formlabs Clear Resin for SLA printing, was performed to validate the reusability of 3D-printed microfluidic chips. Furthermore, the cleaning chip and cleaning procedure proposed here is an efficient and cost- and time-effective solution for enabling the reusability of microfluidic devices. 3D printing is a low-cost, rapid-prototyping method for fabrication that is simpler, cheaper, and faster than soft lithography. The reusable design of this 3D-printed microfluidic chip also replaces the need for fabrication and disposal of many separate devices for the delivery and testing of multiple samples.

As the proposed cleaning chip design has shown favorable results, i.e., minimal protein cross-contamination and protein absorption has occurred, our work here sets a precedent for considering reusability when designing 3D-printed microfluidic devices. Future research directions may look to further assess other factors contributing to the reusability of devices. It is known that the nature of the sample, the materials from which the device is fabricated, and the flow of samples can all contribute to the fouling that occurs. Other 3D-printed materials may be studied to determine their respective absorption characteristics. BSA, a protein often found in urine, was chosen as a representative protein sample; however, different mixtures of proteins may exhibit different absorption characteristics based on charge, hydrophobicity/hydrophilicity, or other surface interactions. With different constituents in the mixture, proteins may unfold; for instance, a protein may be hydrophilic on the surface, but upon unfolding, it might expose a hydrophobic interior, which can affect its absorption behavior. Additionally, since bacteria can feed on residual samples left in the chip and can further contribute to the fouling problem, bacteria adsorption and cross-contamination studies may be performed. It is important to quantify and characterize the bacterial contamination as bacteria could negatively impact the results of some assays. Other future work may also consider the influence of flow rate. Finally, in order to achieve the goal of integrating the demonstrated cleaning chip into a functional microfluidic chip, in the future, the cleaning procedure should be automated and self-contained.

## Figures and Tables

**Table 1:** Analysis and comparison of print orientations. A 500 µm channel height chip was printed using the four different possible orientations: the default angle, vertical, horizontal (along the chip’s side), and flat (face-down). The 500 µm channel height was chosen for this quantification since it was at the threshold of printability. The channels of the chip were injected with red dye, and the fraction of the channel that was open was measured and recorded. For all values presented, there was measurement error of ±0.03 (1 mm measure per 38 mm channel length). The outlet channel consistently had inferior results due to its further distance from the build platform during printing. Vertical and the default angled orientations provide the best results, as evidenced by the highest average channel success values.

Print Orientation	Inlet (Left)	Inlet (Middle)	Inlet (Right)	Outlet	Average Printing Success
Default (45°)	0.83	0.57	0.57	0.03	0.5
Vertical	0.83	0.73	0.67	0.13	0.59
Horizontal	0.5	0.07	0.17	0.07	0.2025
Flat	0	0	0	0	0

**Table 2:** Measurements and analysis of printing success of the final 3D-printed microfluidic chip design. The expected width of all channels was 1000 µm. For all values presented, there was a measurement error of ±0.01 µm. The actual printed width and percent error are presented.

Channel	Printed Width (µm)	Percent Error (%)
Outlet	924.73	7.53
Inlet (Left)	946.25	5.38
Inlet (Middle)	913.98	8.60
Inlet (Right)	892.47	10.75
Average	919.36	8.07

## Supplementary Materials

The following is available online at http://www.mdpi.com/2072-666X/9/10/520/s1, Figure S1: Design iterations of the 3D-printed microfluidic chip for reusability assessment.
