# Supplementary material for: Assessing the Reusability of 3D-Printed Photopolymer Microfluidic Chips for Urine Processing
Source: Micromachines (Basel). 2018 Oct 15;9(10):520. doi: 10.3390/mi9100520 (PMC6215198; doi:10.3390/mi9100520)
Supplement: Supplementary file 1 [file micromachines-09-00520-s001.pdf]

Supplementary Materials: Assessing the Reusability of 3D-Printed Photopolymer Microfluidic Chips for Urine Processing

Eric Lepowsky, Reza Amin and Savas Tasoglu

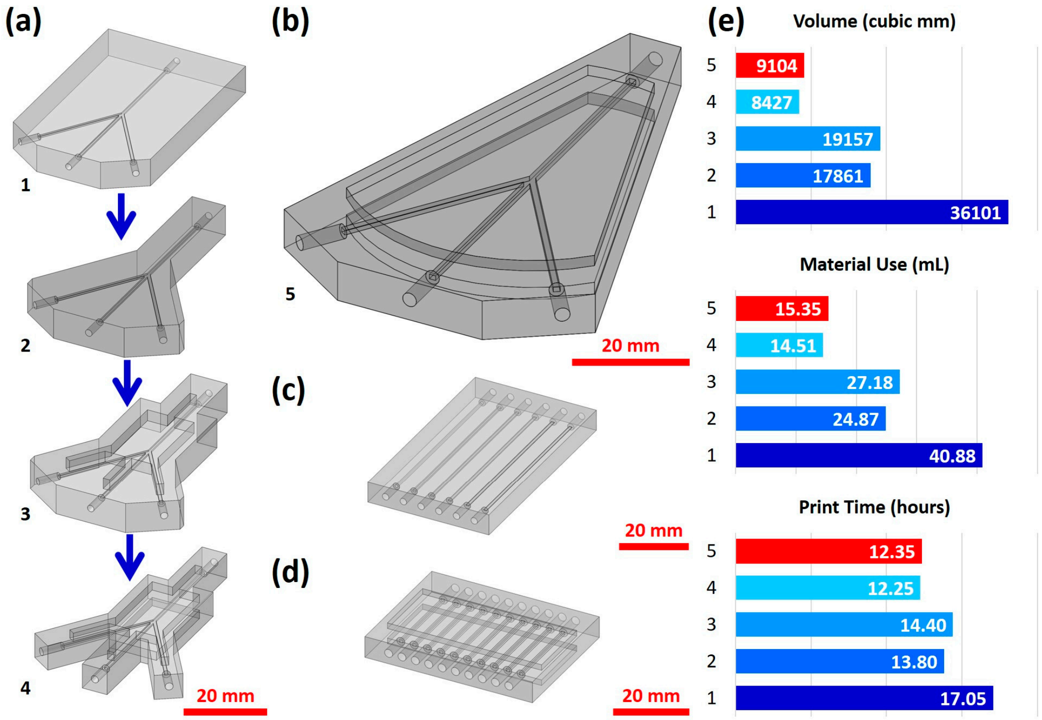

Figure S1. Design iterations of the 3D-printed microfluidic chip for reusability assessment: **(a)** Progression of design iterations of the 3D-printed microfluidic cleaning chip. Preliminary chips, from top to bottom, included a traditional PDMS-style design, reduced outer dimensions to save material, the addition of a viewing window on the top of the chip to improve imaging, and the addition of viewing windows to the top and bottom of the chip for improved through-chip fluorescence imaging; **(b)** Final chip design, with channel dimensions of 1000 µm wide and 750 µm high, features overall thinner design with enlarged viewing windows. These viewing windows are a reduced thickness from the channel to the exterior face of the chip to improve clarity and visibility; the windows were subsequently filled with a thin layer of PDMS; **(c)** Parallel-channel chip used to determine printable channel height. Results showed 500 µm was the minimum to print but with low repeatability; final chip design implemented 750 µm channel height; **(d)** Calibration chip used for chip protein uptake quantification. All channels were the same dimension as the channels of the final chip design; **(e)** Quantification of improvements in the chip design (shorter bars are better). The volume of the chip, the amount of resin used in printing, and the print time are shown for each design iteration.
